# Assessment of movement quality in robot- assisted upper limb rehabilitation after stroke: a review

**DOI:** 10.1186/1743-0003-11-137

**Published:** 2014-09-12

**Authors:** Nurdiana Nordin, Sheng Quan Xie, Burkhard Wünsche

**Affiliations:** Department of Mechanical Engineering, The University of Auckland, 20 Symonds Street, Auckland, TX New Zealand; Department of Computer Science, The University of Auckland, 23 Symonds Street, Auckland, New Zealand; Faculty of Electrical Engineering, Universiti Teknikal Malaysia Melaka, Melaka Malaysia; State Key Laboratory of Digital Equipment and Technology, Huazhong University of Science & Technology, Wuhan, China

**Keywords:** Robot kinematics, Motion analysis, Rehabilitation robotics, Stroke

## Abstract

Studies of stroke patients undergoing robot-assisted rehabilitation have revealed various kinematic parameters describing movement quality of the upper limb. However, due to the different level of stroke impairment and different assessment criteria and interventions, the evaluation of the effectiveness of rehabilitation program is undermined. This paper presents a systematic review of kinematic assessments of movement quality of the upper limb and identifies the suitable parameters describing impairments in stroke patients. A total of 41 different clinical and pilot studies on different phases of stroke recovery utilizing kinematic parameters are evaluated. Kinematic parameters describing movement accuracy are mostly reported for chronic patients with statistically significant outcomes and correlate strongly with clinical assessments. Meanwhile, parameters describing feed-forward sensorimotor control are the most frequently reported in studies on sub-acute patients with significant outcomes albeit without correlation to any clinical assessments. However, lack of measures in coordinated movement and proximal component of upper limb enunciate the difficulties to distinguish the exploitation of joint redundancies exhibited by stroke patients in completing the movement. A further study on overall measures of coordinated movement is recommended.

**Electronic supplementary material:**

The online version of this article (doi:10.1186/1743-0003-11-137) contains supplementary material, which is available to authorized users.

## Introduction

Stroke patients typically exhibit jagged movements [1] as an evidence of loss of control in their affected side. The robotic interventions aimed at improving these weaknesses through repetitive training incorporating increased use of proximal and distal movement [2] in specifically designed task. With the considerable development of robot-assisted therapy [3], the needs to evaluate the contribution of intervention toward intended result is substantial. Kinematic analysis becomes important, mainly in support to the findings of clinical trial on constraint induced movement therapy (CIMT) which eventually distinguish between active restorative movement and compensatory movement [4]. The in-depth evaluation eventually led to the conclusion that the improvement with CIMT is derived from compensation not restoration.

Robotic interventions can offer kinetic measurements (force and torque trends) and electrograms (such as EEG and EMG) to provide further insights on the improvement of the upper limb which is especially important in targeted and perturbed evaluation task. However, kinematic parameters are also substantially used to provide an objective movement evaluation as well as reflections of reduced dynamic behavior. Even though studies have outlined the suitability of kinematic measurements in patients for all phases of stroke recovery to describe bodily function [5], little attention has been made to evaluate the vast variety of kinematic parameters used in current robot-assisted rehabilitation researches particularly to the suitability of the said parameter to significantly capture the changes intended in subjects. Rather, studies on the effectiveness of the rehabilitation robot itself are conducted [3, 6–8] to demonstrate their capability to improve motor function. Particularly, Kwakkel et al. reveal that the review is unable to delineate the difference between genuine improvement of motor restoration and compensation strategies by proximal control of trunk and upper limb [3] after completing the rehabilitation program. They further recommend that the assessment should focus on kinematic analysis as parameters reported through clinical assessments chosen by the researchers in their review are either incomplete or limited to comprehensively evaluate the improvement in patients.

Hence, this study reviews the kinematic parameters adopted by researchers in previous robot-assisted clinical trials and pilot studies at various phases of recovery and attempts to cluster them according to the aspects of movement quality that describes impairment affecting stroke patients. The task in which the measurement is taken place is also evaluated to reveal the context of the assessment and its significance to measurements taken. On the basis of the significant improvement shown by patients in kinematic parameters under study, the suitable parameter is proposed to reflect the specific aspect of movement quality.

## Methodology

### Literature search

The literature search was restricted to English-language articles published between January 2000 and June 2013 in the following electronic databases: PubMed, Web of Science, IEEE Xplore, ScienceDirect, MEDLINE (OvidSP), CDSR (Cochrane database of systematic reviews), Scopus, Compendex, Wiley Online Library, Academic Search Premier, and SpringerLink. The electronic search terms were *Kinematic* AND Robot* AND *(stroke* OR *“cerebrovascular accident”* OR *CVA)*. A free search in Google Scholar and the references listed in primary findings was also conducted to encapsulate wider context. All studies utilizing kinematic parameters in robot-assisted intervention on stroke patients are evaluated. Studies that reported kinetic or biometric parameters accompanying kinematic parameters are included however only kinematic parameters are evaluated. This review specifically excluded the robot-assisted intervention for the hand to focus on both proximal and distal measurements of gross movements. A total of 41 studies in robot-assisted rehabilitation for stroke patients are reviewed for this paper and the parameters obtained are categorized according to the aspects of movement quality as explained by the original authors of the studies.

### Terms and definition

**Figure 1:**
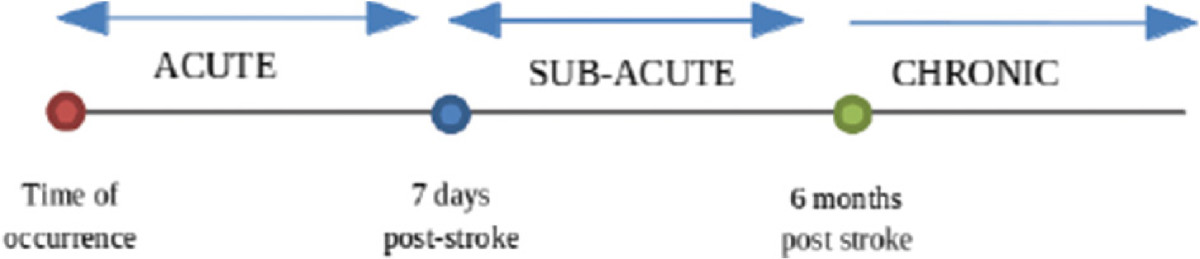
**The continuum of stroke recovery stages.**

Throughout the review, the terms acute, sub-acute, and chronic refer to phases of stroke recovery. The time frame as summarized in Figure 1 follows the recommendation of Sullivan [9] and previous studies of stroke rehabilitation [10–16] in which the stages are defined along a continuum starting on the stroke onset until years post-stroke.

The terms *ipsilateral* and *ipsilesional* are interchangeably used by the authors in the studies reviewed in this paper to refer to the **unaffected** side of the upper limb as stroke patients typically suffer hemiplegia on the opposite side of the brain lesion. However, studies have claimed that the unaffected side also suffers from weaknesses in comparison to healthy person [17].

The term *contralateral* and *contralesional*[14, 18–22] conversely, refer to the **affected** side of the upper limb where the decrease of movement quality is commonly observed. Furthermore, the term proximal and distal are commonly used to explain the segments of the upper limb that are trained by the robot in the studies reviewed. Both terms are defined with respect to the trunk and therefore would refer to the shoulder girdle and arm (proximal) as well as forearm and hand (distal) respectively. Extending the same circumstances, the term unimanual refers to activity performed using one hand, while bimanual refers to activity performed with both hands. The term *hand* in this review refers only to the rigid body without keeping into account its degrees of freedom.

### Method of determining aspects of movement quality

The decreased quality of movement in stroke patients is identified as due to paresis, loss of fractionated movement, abnormal muscle tone and loss of somatosensation [23]. Paresis resulted in a slower, less accurate and less efficient hand movement compared to healthy individuals while the loss of fractionated movement is apparent in abnormal synergy of upper limb segments. Abnormal muscle tone exhibited a jagged movement in which resistive effect of hypertonicity abstain a smoother movement as observed in healthy person whereas the loss of somatosensation affect ability to monitor and correct movement. Impairments are quantitatively measured by various clinical and bio-mechanical methods. Kinematic analysis identifies these weaknesses by end-point analysis [24], inter-joint (intra-limb) coordination [25] and sensorimotor analysis [20]. Besides movement kinematics, the kinetic and biometric aspects such as torque and force trends at selected joints to evaluate abnormal muscle tone [26] and paresis [27], and the use of EMG signal [10, 28, 29] to diagnose the muscle co-activation during movements are also reported. However, due to the scope of this study, kinetic parameters and electrograms will not be discussed further.

Kinematic analysis in stroke patients undergoing conventional treatment has previously revealed the range and coordination of upper limb joints [30, 31], as well as discriminate between compensatory movement and motor recovery [4]. It offers minute details of patient’s movement in contrary to clinical assessments which are developed on the basis of evaluating conventional rehabilitation. As a result, the scores in clinical assessments are highly coarse and ordinal [32, 33] albeit accompanied with rubrics to explain the measures; thus require strong inter-rater reliability score to truly judge the psychometric aspects of the assessment [34]. The fact that the gold standard of clinical assessment remains subjective, helps to alleviate the importance of in-depth analysis and objective measurements to enhance understanding of patient’s improvement by offering a finer level of granularity. However, without comprehensive studies in establishing relationship of a large variety of kinematic variables to aspects of evaluation in standard clinical assessments, the acceptance of kinematic evaluation scales in practice is challenging. Attempts to develop such scale has been made [35] although with minimal success.

Hence, this study attempts to cluster the kinematic parameters adopted by researchers in previous robot-assisted clinical trials and pilot studies at various phases of stroke patients’ recovery according to the aspects of movement quality [refer Additional file 1] to reflect their importance in outlining the four weaknesses affecting the movement. By utilizing suitable kinematic parameters to evaluate rehabilitation treatment, the improvement of specific phase of stroke patients can be better understood and inferred; as whether the improvement is genuine or otherwise. Parameters defining movement planning and inter-limb coordination are clustered together to reflect measures of feed-forward somatosensory loss [36], while temporal efficiency, accuracy and efficacy reflects both the loss of somatosensation (feedback) [18] and paresis [23]. The loss of fractionated movement is associated with parameters measuring intra-limb coordination and task efficiency [37, 38] while the jagged movements due to abnormal muscle tone are associated with parameters defining joint range limits [23], as well as ease and smoothness of movement [1].

Significant outcomes recorded through statistical inference in original article are taken as the ability of the parameter to gauge the changes in stroke patients upon completion of the rehabilitation program. Thus, parameters with significant results (typically with p-value <0.05 in statistical significance test) are considered able to gauge changes in movement quality for the respected stroke population. Furthermore, parameters which are reported to have significant correlation with any of the existing standard clinical assessments are considered to have direct influence to the patients’ clinical outcomes [10, 39]. Additionally, the evaluation activity is also taken into consideration to provide the context of kinematic parameters appraised. The details of the rehabilitation robots have been summarized elsewhere [40–42].

## Integral aspects of robot-assisted therapy

The following subsections elaborate the factors that contributes to the horizons of assessment parameters obtained in this study. Evaluation activities have certain objectives that shaped the kinematic analysis whereby the type of robots, its controller, possible therapy variation as well as their dynamic characteristics influence the range and accuracy of the parameters as measure of true performance of upper limb movements.

### Evaluation activity in robot-assisted therapy

Assessment of stroke patient’s movement has been reported from robot-assisted rehabilitation studies from various evaluation activities. Reaching task is generally chosen because it is the fundamental component in many activities of daily living, requires inter-joint coordination and extensively studied to understand upper limb movements [43].

**Figure 2:**
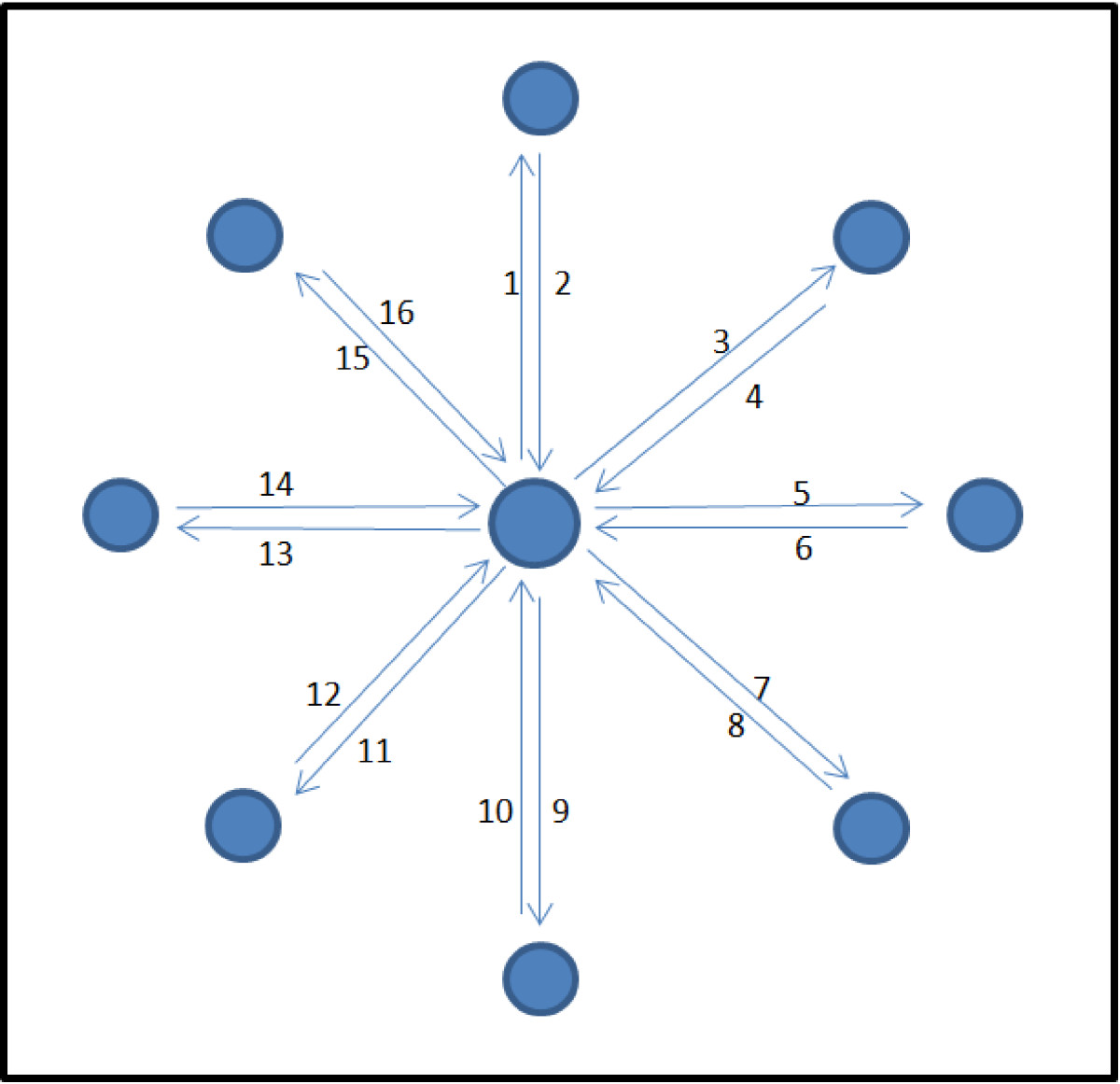
**Center-out point-to-point movement adapted from Rohrer et al.**[1]**.**

While standard clinical assessment such as Fugl-Meyer Assessment (FMA) and Chedoke McMaster Stroke Assessment (CMSA) incorporate free and targeted reaching tasks, evaluation task in robot-assisted therapy typically follows rehabilitation task such as the center-out point-to-point (CO-PTP) reaching activity. The task as illustrated in Figure 2 requires subject to move from center position to a target; then return to the center before beginning to reach the next target, usually situated in circular pattern at a certain radius from the center point.

There are studies that utilized different evaluation task in comparison to the rehabilitation task [10, 39] to insinuate that the training can be generalized to untrained activity in the same workspace. The evaluation activity has certain emphases on aspects of movement quality that the studies claimed to measure. Table 1 summarized the findings. Based on the activities reported, the aspects of movement quality that can be observed indeed depend on the type of evaluation task performed. Hence, the combination of untrained task within the trained workspace, with the multi-level target to defy gravitational influence as well as task that challenges the range of movement from the area of unaffected to affected side may provide greater insights to the aspects of movement quality in stroke patients.

**Table 1:** **Overview of the evaluation activity performed in robot-assisted rehabilitation**

Evaluation activity	Body plane	Evaluation objectives	Aspect of movement quality addressed	Studies
Center-out point-to-point (CO-PTP)	Transverse	Feed-forward and Feedback control	Temporal efficiency, Ease, Smoothness, Accuracy, Planning, Efficacy, Movement efficiency, Inter-limb coordination, Range	[1, 18, 20, 44–49]
	Frontal	Feedback control, Gravity-compensation	Temporal efficiency, Smoothness	[50, 51]
Point-to-Point Reaching	Transverse	Feed-forward, Feedback control, Perturbation- compensation	Temporal efficiency, Ease, Smoothness, Planning, Movement efficiency	[10]
	Sagittal/Frontal	Range of motion, Feed-forward and Feedback control, Gravity-compensation	Planning, Temporal efficiency, Smoothness, Range	[22, 22, 45, 52–59]
Free/Constrained/Targeted Reaching	Sagittal/Frontal	Range of motion, Perturbation-compensation, Feed-forward and Feedback control, Gravity-compensation	Planning, Temporal Efficiency, Range, Smoothness, Movement Efficiency	[29, 60]
Shape drawing	Transverse	Untrained activity, synergy	Accuracy, Intra-limb coordination	[39]
Shape tracing/tracking	Transverse	Synergy, Feedback control	Accuracy, Efficacy, Ease, Smoothness	[13, 17, 21, 27, 59, 61, 62]
	Frontal	Synergy, Feedback control	Ease, Accuracy	[63]
Bimanual matching	Transverse	Somatosensory (Proprioception)	Planning, Movement efficiency, Ease	[19, 20, 64]
Bimanual reaching	Sagittal	Somatosensory, Coordination	Inter-limb coordination, Efficacy, Ease	[59, 65]
Isolated movement	All	Range of motion	Range	[66–68]
Activity of daily living	All	Functional ability	Inter-limb coordination, Temporal efficiency	[7]
Virtual games	All	Functional ability	Range	[67, 92]

### The influence of rehabilitation robots to assessment ability

Rehabilitation robots that are considered in this study are of two different types; end-effector and exoskeleton. Robots such as MIT-MANUS, InMotion2 and MIME are of end-effector type. Typically forearm is supported by these robots and forces are generated at the interface to assist the movement of the patient. Conversely, exoskeletons such as EXO-UL7, Armeo and ARMin support both arm and forearm which enable controlled torque application to multiple interface of the upper limb. Table 2 summarizes the variation of rehabilitation robots that are taken into account in this study.

**Table 2:** **Overview of the rehabilitation robot included in the review**

Rehabilitation robot	Structure	Supported	Controller	Possible therapy	Range of motion	Gravity-compensation	Back-drivability
		segment		variation			
MIT-MANUS	2DOF (end-effector)	Forearm	Impedance control	Passive, Resistive	Planar movement	None	Yes
InMotion2	2DOF (end-effector)	Forearm	Impedance control	Passive, Resistive, Assist-as-needed	Planar movement	None	Yes
InMotion3	5DOF (end-effector)	Forearm	Impedance control	Passive, Resistive, Assist-as-needed	3D movement	None	Yes
ARM-Guide	2DOF (end-effector)	Forearm	Impedance control	Passive, Resistive	Constrained linear movement	Yes	None
MIME	pair of 3DOF (end-effector)	Forearm	Impedance/Admittance control	Passive, Active-assisted, Active-constrained, Bimanual	3D movement	Yes	None
Bi-ManuTrack	2DOF (end-effector)	Forearm	Not specified	Bimanual active, Bimanual passive, Bimanual single active	Planar movement	None	None
Bilateral force-induced isokinetic arm movement trainer (BFIAMT)	2DOF (end-effector)	Forearm	Admittance control	Bimanual passive, active-passive, resistive, reciprocal, symmetric	Planar movement	None	None
Braccio di Ferro (BdF)	2DOF (end-effector)	Forearm	Impedance control	Active, Active-resisted, Resistive	Planar movement	None	Yes
REHAROB	two 6DOF robot (end-effector)	Arm, Forearm	Admittance control	Moevement at constant low velocity	3D movement	Yes	None
Uni of Guelph Therapeutic Robotic System (CRS-Robotics)	5DOF (end-effector)	Forearm	Impedance control	Active, Passive, Active-assisted	3D movement	None	Yes
MACARM	6DOF (end-effector)	Arm/Forearm	Impedance control	Gravity assistance	3D movement	Yes	None
MEMOS	2DOF (end-effector)	Forearm	Admittance control	Passive, Active, Active-assisted	Planar movement	None	None
HapticMASTER/ADLER/BiAS-ADLER	3DOF (end-effector)	Forearm	Admittance control	Active, Active-constrained, Drink and pour	3D movement	None	Yes
KINARM	2DOF (exoskeleton)	Arm, Forearm	Impedance control	Active-resisted, Bimanual Matching	Planar movement	None	Yes
L-Exos	5DOF (exoskeleton)	Arm, Forearm	Impedance control	Impedance assistance, gravity assistance	3D movement	Yes	Yes
EXO-UL7	two 7DOF (exoskeleton)	Arm, Forearm	Neural control	Master-slave bimanual active guidance, unimanual active guidance	3D movement	Yes	Yes
T-WREX/ArmeoSpring	5DOF (exoskeleton)	Arm, Forearm	Impedance control	Passive	3D movement	Yess	None
ARMin/ARMin II/ARMin III	6DOF (exoskeleton)	Arm, Forearm	Impedance/Admittance control	Passive, Active-assisted, Resistive	3D movement	Yes	Yes

Indeed, the robot characteristics, its degree of freedom and control strategy have the influence on the range of parameters that it can provide. End effector robots are typically developed to assist planar movements with the exception of systems such as REHAROB and MIME that are supported by industrial robots which have greater degrees of freedom. Furthermore, they are not able to provide the range of movement of upper limb joints such as shoulder and elbow angle using internal robot measures, thus proximal assessment such as intra-limb coordination which is beneficial to understand the interaction of upper limb components have to be inferred on the end-effector quality of performing synergistic tasks [39] such as circle drawing and shape tracing.

Exoskeleton robots on the contrary are built side-by-side with the upper limb which provides isolated joint control and greater range of assessment parameters as proximal segments are being interfaced to the system. However, precise coupling of the robot kinematics and upper limb kinematics are required for the internal robot measurement to be feasible. This means that the transformation of kinematic parameters in robot functional frame to anatomical frame should be available or at least controlled during assessment session for a useful clinical interpretation. This can be realized by designing specific joint configuration that deemed the robot as statically determined [67, 69] and provide system of linkages that allow the movement of anatomical segment’s center of rotation as the movement occurs [70].

The control scheme of the rehabilitation robot plays an important role in providing assessment data. While impedance controlled robots such as MIT-MANUS/InMotion and ARM-Guide offer stable dynamic interaction with stiff environment such as in the case of targeted movement and shape tracing, report have shown that even low-impedance end-point movement is susceptible to robot’s intrinsic dynamics [71]. The consequence is remarkably consistent 2D surfaces emerged from trial-to-trial and between subjects which would affect the ability of the robot to provide meaningful assessment. In contrast, admittance controlled robots such as MIME and ARMin has high level accuracy and impart negligible amount of inertia during free reaching task. However, to accommodate the complexity, the system for example employs harmonic drive actuators [69] where considerable friction exists when the robot is in passive state. Thus, assessments are realized during counterbalanced (transparent) state which therefore relies on the performance of the robot’s controller to distinguish user’s performance from the influence of robot dynamics.

Beyond the robot structure, the possible therapy variation may influence the range of assessment data provided as well. While passive assessment session requires backdrivability of the robot, user’s share of control in active-assisted and resistive rehabilitation session can be beneficial for continuous assessment. It is important to emphasize however, that the robotic system must be able to distinguish the user’s contribution during the therapy from the sum of external forces which includes gravity, inertia, centrifugal and Coriolis forces, passive mechanical forces and forces related to muscle activity [72].

In summary, it can be concluded that optimal assessment data can be provided solely by the robot without external motion capture when no perturbation either from internal dynamics of the robot or gravitational loading is guaranteed and the kinematic coupling between the robot and user is controlled.

## Kinematic parameters evaluating movement quality

**Figure 3:**
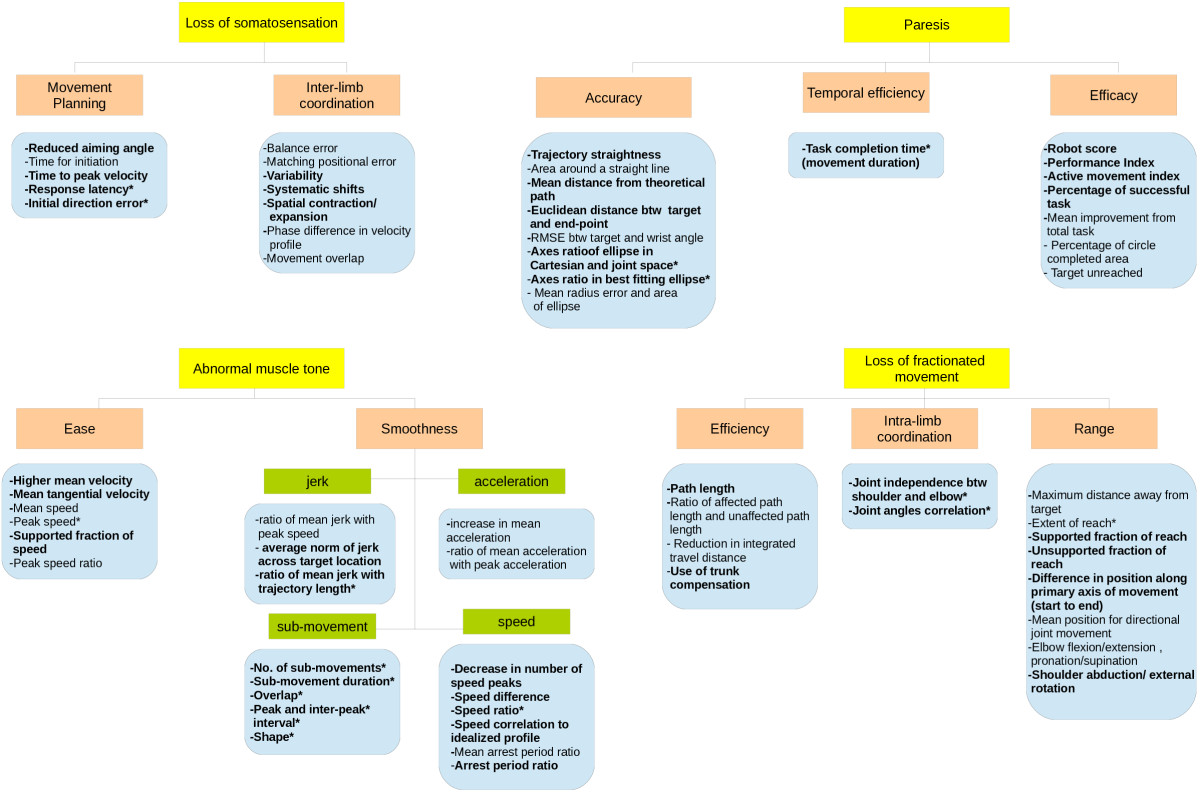
**Overview of parameters used for kinematic assessment in robot-assisted upper limb rehabilitation.**

The assessment conducted in studies of robot-assisted rehabilitation reviewed in this paper generally focuses on end-point movement except for parameters defining joint range limits, intra-limb and inter-limb coordination. The following sub-section offers the in-depth explanation of parameters symbolized and segregated based on the qualitative aspects they represent [refer Additional file 1]. Figure 3 provides the overview on the 10 aspects of movement quality addressed in subsequent sections.

### Movement planning

The extent of task planning in stroke individual is attributed to the feed-forward sensorimotor control, in which previous studies reveals that predictability of the target influences the strategy to attain them [73]. The sixth column listed all the studies [refer Additional file 1] that utilize kinematic parameters to reflect planning extent of stroke patients. Changes in direction, the time taken for the initiation and the initial speed of subject’s endpoint are parameters chosen by researchers in this review to reflect feed-forward sensorimotor control.

Zollo et al. [10] describe reduced aiming angle in which angular difference between target direction and direction of travel is calculated from starting point up to peak speed point [74]. Significant results are recorded for chronic patients in unperturbed and resistive PTP activity [10], CO-PTP activity [18] as well as multi-level PTP activity [45], suggesting that the parameter is suitable to gauge capability of chronic patients in planning to overcome external perturbations, changes in direction and gravitational influence to reach the target. Furthermore, the parameter is significantly correlated to Fugl-Meyer Upper Extremity portion (FMA-UE), Motor Power (MP) [10, 35] and Motor Status Score (MSS) although not to Modified Ashworth Scale (MAS) [35]. The correlation to FMA-UE and MSS indicates that the reduced difference in target direction reveals improvement in motor synergy and voluntary activities. Since MSS is developed to augment FMA scores in specifying voluntary movement in sub-acute patients, the consistent result is expected. Correlation with MP signifies that reduced aiming angle also reflects the increase in strength in isolated muscle group. However, the lack of correlation in MAS and CMSA scores results in parameter’s incapability to differentiate level of hypertonia and Chedoke stages of impairment.

Mazzoleni et al. [46] on the other hand, propose the time taken for the initiation to evaluate the extent of planning in which the percentage of the time for movement initiation during the first 2 seconds of each requested movement is recorded. This movement onset measure is done regardless of direction taken without robot assistance. Insignificant results in chronic patients were initially presented [46] however, significant percentage decrease in chronic patients is presented later in a study evaluating both sub-acute and chronic patients [48] in CO-PTP activity which indicates the reduced latency period before starting the directed movement at the end of rehabilitation program. Iwamuro et al. [58] similarly report significant decrease in time to peak speed in multi-level PTP which signifies that the reduced latency parameter is able to gauge planning changes in different direction and gravitational influence.

In bimanual evaluation, Chang et al. [60] suggest the use of peak velocity along with percentage time to peak velocity to reflect movement planning in symmetric bimanual movement and report significant within-subject effect in both parameters respectively. Response latency (RL) and initial direction error (IDE) [19] in bimanual matching study outline almost half of the left-affected patients to be significantly out of normative control range. Exemplar data from a stroke subject shows significant difficulty with initiation of matching movements and high variability in RL. Both parameters are also reported to correlate significantly with Functional Independence Measure (FIM) while IDE correlates strongly to Purdue Peg Board (PPB), CMSA and Thumb Localizing Test (TLT) as well. Dukelow et al. employed the study of CO-PTP movement and bimanual matching to determine the relationship between kinematic parameters used to analyze both task and reported the use of postural control, initial directional error and reaction time in the unimanual task [20]. Although all parameters show statistical categorical result, none of the parameters demonstrates significant correlation to any matching evaluation parameters or abnormal execution. This indicates that planning strategy in unimanual task does not translate to bimanual activity.

Based on the reported clinical results, kinematic parameters that define reduced deviation in target direction and response latency are appropriate to measure the extent of feed-forward sensorimotor control in the sense that the improvement reflects both dimensions (time and direction) to reach the target intended. However, the end point measurements do not confirm whether such improvement is a genuine motor recovery or due to the appearance of compensation, as proximal control is not taken into account. The attempt to use force directional error [46] in end point measurement to reflect compensation strategies also does not reveal the influence of proximal control. While it is crucial to differentiate the improvement whether it is purely distal or proximal or both [3], the uncertainty is apparent as no specific measures are taken to differentiate them. Therefore, it is suggested that future studies measure the extent of planning in stroke patients in both segments to better distinguish the cause of the improvement. Where the application permits, the bimanual evaluation might suggest further details as the result presented in such activity suggests that unimanual assessment of movement planning does not extend to bimanual functions.

### Inter-limb coordination

In the bimanual matching task measuring proprioception, the measure of inter-limb coordination is reported through studies assessing the accuracy of the position sense. Sanguineti et al. outlined the balance error [59] in bimanual forward/backward movement using T-bar attached at the end effector as measures of coordination and reported an improvement in chronic patients albeit without statistical inference. Squeri et al. [49] in their proprioceptive training utilized matching positional error at movement termination as measure of coordination of hand position sense in a single case study. They further scrutinize the recorded position into medial/lateral and anterior/posterior shift and skew as well as shrink coefficient on polar lattice of test points in CO-PTP movement. Considerable large shifts in anterior/posterior and smaller shrink coefficient are recorded in comparison to healthy control.

Dukelow et al. [64] in the same training utilize three measurement of coordination in hand position sense: variability, systematic shifts and spatial contraction/expansion. Relative to the healthy controls, both left-affected and right-affected sub-acute stroke subjects, showed greater variability matching with their unaffected arm. Left-affected subjects displayed significantly higher variability than right-affected subjects when matching with their unaffected arm. Stroke subjects also exhibited greater mean systematic shifts than controls matching with their unaffected arm. All subjects reported a greater spatial contraction than the controls but there was no significant difference between the two stroke groups. In later study [20], they confirmed the categorical relationship of all the parameters and reported the insignificant use of single parameter to distinguish the stroke patients from healthy subjects.

Johnson et al. [11] utilized three measures of inter-limb coordination; velocity profile of both hands, the phase difference and movement overlap. The increase in relative phase metric (the lag between right and left limbs) indicates lower inter-limb coordination. In the drink task, the velocity profile of the chronic stroke subjects did not remain in sync while controls exhibit highly symmetric movements. The average %MO decreased significantly for chronic stroke subjects when compared to able-bodied subjects while differences in phase difference are not significant. In pour task, movement of stroke patients were not distinctive as opposed to two distinct bell shaped movements for dominant and four for the non-dominant hand of the healthy subjects. Both phase difference and movement overlap however did not show significant changes in comparison to healthy subjects.

The extent of feed-forward sensorimotor control in bimanual task is evident through measures of position sense [64], however researchers have proposed two different dimension of position sense, through planar and activity of daily living (ADL) task. While it is tempting to utilize ADL task for evaluation, planar task provides greater insights and thus recommended for evaluation.

### Temporal efficiency

Temporal efficiency defines the optimal time taken to complete the task and defined as the time to perform a certain activity or movement, elapsed from movement onset and movement termination [10]; and the time taken is expected to reduce with patient’s recovery.

Researchers apparently reported variations of definition in determining the movement onset and movement termination thus varying the movement blocks that are taken into account for analysis. The movement onset [10, 19, 46–48] is defined as the time instant where velocity exceeds a threshold of 10% of peak velocity and movement termination as time instant where velocity goes below a threshold of 10% of peak velocity based on Smith et al. definition [74, 75]. Dipietro et al. [39] however arbitrarily considered a 2% threshold whereas Frisoli et al. [50] and Johnson et al. [11] define them as a 5% threshold of the maximum velocity. While other researchers identified single velocity threshold for both movement onset and end, Coderre et al. [18] suggest statistical threshold based on hand speed to account for different phases of stroke patients. However, out of these definitions, the researches that commit to onset and offset at 5% of maximum velocity are the only studies that utilize movement duration as their index of performance and presented significant changes.

Contradicting results are reported from gravitationally influenced task. Statistically insignificant difference in task completion time [54] was reported using ARM-Guide in reaching along linearly inclining track at the lateral side of the arm and multi-level target involving shoulder abduction in a diagonal pattern away from the body [45]. However, Lum et al. [55] reports a significant decrease in movement time in multi-level targeted reaching involving forward-medial (shoulder flexion/adduction) and directly forward (shoulder flexion) targets; however not for forward-lateral (shoulder flexion/abduction, external rotation), and directly lateral (abduction/external rotation) targets. In CO-PTP performed in sagittal plane, Frisoli et al. [50, 51] also reported significant decrease in total duration for ipsilateral target. This suggests that temporal efficiency can be significantly captured in location closer to the center and ipsilaterally across the body than others.

In a transverse plane CO-PTP activity, Conroy et al. [45] reported statistically insignificant changes in movement duration. However a progressive reduction is recorded in unperturbed and resistive PTP activity in free space [10] which suggests that movement duration is sensitive in planar evaluation where the target is not restricted. Movement times are also significantly longer in sub-acute subjects [18] with left-affected patients perform poorly in comparison to healthy controls and right-affected subjects which suggested that lesion area also influences the temporal efficiency.

In bimanual assessment of chronic patients, significant within-subject effect in movement time [60] is reported for bimanual symmetric arm push and pull movements. Johnson et al. [11] also reported a significant decrease in bimanual pour and drink task in comparison to healthy subjects.

Significant correlation of movement duration to FMA-UE, MP, and MSS except MAS are reported and consequently becomes one of the predictor in FMA-UE and MSS score after backward regression analysis [35]. This is to be expected since MSS is built based on FMA-scale and employ finer grading for isolated movement and evaluates complete range of motor function in upper limb [76]. MP however is derived from MAS with standardized guidelines which might be the determining factor that signify the correlation with the parameter.

Thus, clinical studies in stroke patients summarize that a measure of temporal efficiency should be pre-empted with the definition of lesion especially in sub-acute population as to minimize the improvement bias due to the side of the lesion. To better gauge the improvements, it must be evaluated in targets located ipsilateral to center of the subjects’ body if gravitational influence is concerned while planar evaluation should consider resistive task in free space. The lack of correlation with MAS scale might suggest that the parameter will not be able to distinguish improvements made by subjects if velocity-dependent task is evaluated.

### Movement accuracy

The accuracy of movement is reported mainly in literature as straightness which is the measure of end-point trajectory error relative to straight line. The importance of this measure is reported by Cirstea et al. [77] such that the degree of movement accuracy is significantly correlated with severity of clinical symptoms. Significant improvement in straightness is reported in multi-level PTP activity [14, 54] and in CO-PTP [46]. Similarly, Kim et al. [16] utilize area around a straight line in assisted and unassisted virtual PTP game in which better results are recorded by unimanual group against bimanual group. Panarese et al. [62] further elaborate that significant improvement of straightness is influenced by target direction.

Other researchers opt to use the theoretical path of the task or location of the target as the reference instead of a straight line. Colombo et al. [61] defines movement accuracy as mean absolute values of distance of each point of the path from the theoretical path in which the recorded values closer to zero indicates higher accuracy in shape tracing activity. Significant improvement in chronic patients performing shape tracing is reported [13, 27, 63] although not with sub-acute patients [13, 78]. Abdullah et al. [78] further explain that smaller offset is recorded mostly in circular test but greater offset is recorded in square test with patient exhibiting greater offset in circular test also exhibit greater offset in square test.

Similarly, Daly et al. [44] utilized 2D Euclidean distance between target and the subject’s end point and reported a statistically significant gain in CO-PTP activity. Hu et al. [79] employed root mean square error (RMSE) between the target and the actual wrist angle during cursor tracking activity and found a significant decrease in the first 7 session. However, no further significant variation is reported in the subsequent session. Based on previous study in motor learning [80], small or static progress reflect the learning of a skilled movement. The author claimed that the post stroke motor recovery was similar to motor learning to some extent, and what was known about motor learning might predict the course of motor recovery [21]. Thus after session 7, when there was no further significant decrease in the overall RMSE value; the wrist tracking skill could be regarded as stably learned by most of the subjects.

Researchers also combined the measure of straightness with measure of ellipticity to capture the relation of accuracy to circular trajectory. Axes ratio in both Cartesian space and joint space [39] are evaluated and reported with improvement mainly from significant changes in minor axis in Cartesian space. Axes ratio in joint space also increases significantly at discharge. Both parameters are significantly correlated to FMA synergy portion and FMA total score; however a decrease in correlation is apparent from initial to discharge signifying that the improvement in axes ratio might not reflect the same recovery context as the FMA score. Similarly, Bosecker et al. [35] also reported that the axes ratio of the best fitting ellipse in unconstrained circle drawing are significantly correlated to FMA-UE, MP, and MSS except MAS albeit not being the strong predictor for the scales for chronic patients. Sanchez et al. [56] utilized mean radius error area and circularity measure area in measuring ability to trace a circle with and without gravity balance and reported significant decrease in both parameters with gravity balance.

The implication of these findings is that the accuracy measures should be evaluated by shape tracing, where the influence of direction and target location can provide better insights. Measure of ellipticity seems to extend the accuracy results to clinical outcomes, however by conducting them with gravitational influence might provide deeper understanding.

### Movement efficacy

Movement efficacy is the measures defining ability of the subjects to produce intended result, thus it is closely related to the outcome performance of specific intervention. Researchers have opted for task based approach to evaluate the quality of movement as a result of using the device.

Researchers decided to combine several parameters for efficacy as evident in [61, 62]. Colombo et al. [61] utilizes three robotic measures for efficacy; the robot score, performance index and active movement index (AMI). Significant changes are reported in all parameters. Significant changes is also reported later for both robot score and performance index [27] in both group utilizing wrist and shoulder-elbow manipulator however only chronic patients utilizing shoulder-elbow manipulator shows significant changes in AMI. Finally [13], only AMI is utilized as a measure of effectiveness in sub-acute and chronic patients’ for robot-assisted rehabilitation routine. All patients reported to have statistically significant improvement. The authors claimed that assessment of motor efficacy as measures of independence from the device in the task execution which then enables adaptation of the difficulty of the required task to be tailored to the patient’s disability. Similarly, Panarese et al. [62] combine the percentage of successful task derived from a state-space model from measures of number of successful task, speed, number of peaks and distance. They reported significant increase along each segment and the curves are significantly different suggesting the sub-task dependent time-course.

Others however chose task-based single parameter to evaluate efficacy. Squeri et al. [65] evaluated the total number of trial blocks and reported a mean improvement of 3 blocks (out of 10 blocks) at the end of the bimanual training. Sanchez et al. [56] in severe stroke patients, utilized percentage of circle completed area to reflect the efficacy of tracing a circle however produced ambiguous results. Meanwhile, Coderre et al. [18] in visual guided reaching task utilized the ‘no movement end’ as measures of trials where target is not reached or subject did not stabilize at the peripheral target. It is reported as one of significant parameters that left-affected patients perform worse than the right-affected subjects and controls however the parameter did not generalize to all patients.

Studies by Colombo et al. revealed interesting result from the use of AMI score. It significantly reflects changes in efficacy for chronic patients utilizing proximal aid (shoulder-elbow manipulator) than distal aid (wrist manipulator). Furthermore, Panarese et al. also suggested that efficacy of the task relies on the ease of movement, smoothness, accuracy and direction of the task performed. The common point in studies presented however, is that the measures of end-point movements are utilized to build the efficacy parameter rather than the inclusion of a composition of intra-limb coordination. This prohibited the reveal of the underlying influence of whether the efficacy is the result of movement recovery or compensatory strategies adopted by the subjects.

### Movement efficiency

The nature of complex structure in upper limb rehabilitation permits the same end-point movement to be achieved in a number of different ways, reflecting kinematic redundancies [81]. Thus, a measure of efficiency is determined by the most optimal way for the end-point movement to reach the target. Researchers suggested that the shortest trajectory to the target as measure of efficient movement, other trajectories indicate greater effort or the dismal use of other movement strategies to complete the movement. It reflects the greater energy expenditure than normal movement pattern [13].

Significant improvement of path length is recorded in chronic patients undergoing unimanual rehabilitation [10, 13, 18, 27, 57] which reveals a more pronounced impairments in left-affected patients [18] and a strong correlation to the amount of gravity compensation provided [57]. Normalized measure of path length is reported to capture sub-acute population significantly better than chronic patients [13]. Furthermore, the path length ratio is reported to be strongly correlated to MP scale although not with FMA [10]. Target location however did not have significant impact.

In bimanual study, Semrau et al. [19] however opt for a ratio in which the total movement length of the subject’s active arm is divided by the length moved by the passive arm. They report a moderately abnormal matching in chronic subjects and are more variable about the distance they moved to match the movement than healthy control groups. Kim et al. [16] suggest the reduction in integrated travel distance for virtual reality games employed during bimanual against unimanual study as measures of efficiency. Bimanual training group patients are reported to show higher travel distance for most games.

On the contrary of trajectory measurements, others opt for the lack of efficiency through motor compensation to reveal the inefficient movement of the stroke patients. Levin et al. [82] defines motor compensation as the appearance of new motor patterns resulting from the adaptation of remaining motor elements or substitution. In upper limb, the previous literature by the author [83] suggested that the compensation include the use of movement patterns that incorporate trunk displacement and rotation, scapular elevation, shoulder abduction, and internal rotation. Wu et al. [15] uses the ratio of sagittal displacement between the index marker and the sternal marker to the sagittal displacement of the sternal marker as measures of arm-trunk compensation in bimanual and unimanual study against healthy controls. More pronounced trunk compensation is reported in unimanual group while bimanual group elicited larger improvements in reducing compensatory trunk movements in targeted reaching activity.

The choice of kinematic parameters defining efficiency is quite ambiguous in the reviewed studies as optimal movement can be attained with lowest energy expenditure of the upper limb. Thus, the kinematic deficits can be portrayed as the reflection of inadequacy of dynamic interaction of the upper limb. Indeed, movement efficiency cannot be discerned with kinematics measure alone when optimality of redundant system is addressed. In targeted evaluation task in which feed-back control is required, the optimality of the movement towards the target relies on minimization of dynamic interaction torques of arm and forearm due to forearm inertia in accelerating the hand towards the target [84]. Failure to do so results in undesired acceleration of the proximal segment which can be observed by the compensatory trunk and shoulder girdle movement. Furthermore, the involvement of muscle activities in active movements is difficult to be discerned using force alone. For example, incoordination of agonist/antagonist co-contraction might be misinterpreted as weakness in agonist muscles in synergistic evaluation task thus requires EMG measures of muscle co-activation for confirmation [72]? The kinematic parameters can therefore partially provide clinical insights to the patient’s performance during evaluation. However, considering the compensatory movement of the trunk and shoulder girdle that occur during synergistic reaching, the addition of these components might encapsulate better way to represent movement efficiency if only kinematic measures are possible at the time of evaluation.

### Intra-limb coordination

The redundancies in upper limb joints [85] enable the production of different strategies to complete the task, thus severely affected subjects are more likely to impose couplings of joints to complete the task than healthy subjects [86]. Bosecker et al. [35] utilize the degree of independence between shoulder and elbow movement as the measure of joint synergy in unconstrained circle drawing assessment. The circle-drawing task is reported to involve the coordination of both shoulder (horizontal) abduction/adduction and elbow flexion/extension [30]. They reported significant correlation of joint independence to Fugl-Meyer (upper extremity), MP, and MSS and consequently become one of the predictor in FMA-UE and MSS score after backward regression analysis. The measure however is not significantly correlated with MAS.

Dipietro [39] utilizes joint angles correlation to reflect the same idea and reported significant decrease across all subjects from admission to discharge and significantly correlated to FMA-synergy portion and FMA total score albeit with decreasing correlation factor from initial to discharge. This finding implies a better decoupling of shoulder and elbow of the paretic arm at discharge. Kung et al. [28] in their recent studies suggested a dynamic assessment of joint synergy during rectilinear tracking mainly due to the fact that daily activities are dynamic. The contralateral and ipsilateral targets are reported to be more useful for assessing abnormal synergies. Crocher et al. [87] on the other hand use explicit model based on linear relationship between joint velocities. Principal Component Analysis (PCA) is used to determine the constraint in redundancy of pointing task, that is the unused subspace with the least variance and quantify the difference between subject’s natural constraint, applied constraint by therapist and robot’s imposed constraint. It is interesting to note that the measure can detect the kinematic coupling with the first three principal component up to 96.4*%*. Furthermore, the use of therapist constraint restricts the redundancy of the upper limb by decreasing elbow angle without significantly modify the endpoint trajectory. This corresponds to the end goal of normal synergy which is reducing the excessive elbow elevation.

### Range of motion

Task-based evaluation and isolated joint measurement are adopted by researchers to reflect the movement capacity in stroke patients. In gravitationally influenced activity, Kahn et al. [54] utilized a measure of maximum distance moved away from reach start position although reported statistically insignificant changes in chronic patients. However, Lum et al. [88, 89] report a significant improvement in the extent of reach to shoulder-level target in comparison to healthy control subjects. Statistical trends indicated subjects regardless of CMSA stages reach slightly further to ipsilateral targets and for subjects in lower CMSA stages to have more difficulty reaching to higher targets. Thus in later study, a revised measure is proposed; the supported fraction range of movement (FR) along a straight path and the measure of unsupported fraction of range (FRu) for free reaching activity [14]. Significant improvements in FRs for all chronic subjects are reported regardless of different training group or impairment level [58].

On the contrary to the use of distance measures, Ellis et al. [90] propose measures of work area with a total of 9 support levels were randomized for testing. Significant effect of support level to the difference in work area is reported. Post-hoc analysis indicated that there was a significant difference between levels separated by 2 intervals. A positive and significant relationship between the work area and each clinical assessment tested (FMA (shoulder/elbow portion: FMAs, total arm score: FMAt), CMSA (arm portion: CMSa, hand portion: CMSh), MAS and Stroke Impact Scale (SIS)) are reported with the exception of the CMSh, domains 2-6 and 8-9 on the SIS, and the MAS. Participants with very similar or identical scores on both the FMAt and CMSa have a variable range of work area measurements. The significant relationship to FM and CMS indicate the concurrent validity of the parameter while the exclusion of CMSh might indicate the parameter captures the proximal changes rather than distal changes. However, the proximal changes should also be taken with caution since similar score in FMAt and CMSa exhibits variation in work area.

In bimanual assessment, the range is defined by the difference in position along the primary axis of movement from movement start to end in bimanual reaching activities [29]. Significantly albeit slightly further range is achieved by chronic subjects in robot-assisted bimanual planar reaching task (on transverse plane) when the trajectory is defined by unaffected arm in comparison to when the trajectory is defined by the robot while the evaluation on multi-level reaching task does not reveal any significant difference. Significantly larger range is also observed in robot-assisted planar task when the trajectory is specified by unaffected arm in comparison to voluntary movement suggesting that gravitational compensation helps to improve range of movement. Evaluation on robot-assisted vs. voluntary muti-level reaching task also do not reveal any significant difference.

The range measurements of isolated joint are also observed especially with studies related to proving a specific device usability to extend the range of specific joints. In the series of assisted and unassisted CO-PTP movement, Mazzoleni et al. [47] proposed the mean position for north toward-abduction; south toward-adduction; east toward-extension; west toward-flexion as measures for range of wrist movement but improvements are not statistically significant. Insignificant improvement is also reported [66] for elbow pronation/supination and flexion/extension at the end of repetitive and monotonous slow movement therapy. Utilizing the same approach however, yielded significant improvement in active range of elbow flexion though not on active range of shoulder-girdle forward bending [68]. Adopting virtual games for reaching, Simkins et al. [91] reported statistically significant improvement in shoulder abduction and external rotation following bimanual movement training and standard care in isolated joint measures.

Taking into consideration the outcomes of these clinical studies, the unimanual task based evaluation differentiates the extent of reach in gravitationally influenced task better than planar (on transverse plane) task while the bimanual task produces significant results in planar evaluation suggesting that gravitational balance affect unimanual movement evaluation more than bimanual. The isolated joint evaluation reveals that monotonous slow movement therapy may not benefit the improvement in pronation/supination, while targeted reaching may have more influence in proximal segment in comparison to distal.

### Ease of movement

The ease of movement is portrayed as the ability to perform activity as effortlessly as possible. The record of higher mean velocity is generally taken as the decrease of abnormal effort to perform the required movement. In robot-assisted training, the presence of gravity compensation increases the ability of patients to perform the task.It is important to emphasize that the ease of movement relies on the continuous effort of the patient to complete the movement which includes the ability to reduce interaction torques and maintain normal co-activation of agonist/antagonist muscles especially when the timed task is performed. Thus, the use of mean and peak speed to signify ease of movement should be interpreted with caution whenever the force or EMG measurements are unavailable

**Figure 4:**
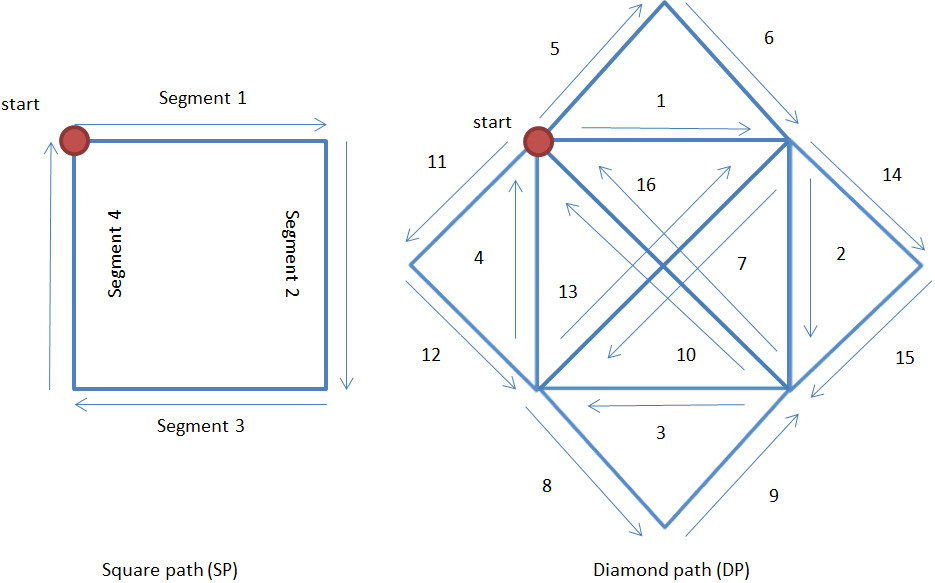
**Point-to-Point movement following square path and diamond path adapted from Panarese et al.** [62]**.** The segments of diamond path are classified to within (1, 2, 3, 4, 7, 10, 13 and 16) and outside trained workspace (5, 6, 8, 9, 11, 12, 14 and 15), proximal (2, 6, 7, 8, 11, 12, 14 and 15) and distal (4, 5, 9, 10, 13 and 16), dominant (3, 7, 10, 11, 15 and 16) and non-dominant (1, 5, 6, 8, 9, 12, 13 and 14).

Colombo et al. [61] reported higher mean velocity with significant increase in the chronic patients utilizing MEMOS and similarly in later study [27] than those utilizing wrist manipulator. Statistically significant increase is reported later [13] for mean velocity in both sub-acute and chronic patient. A significant increase in mean speed at the end of the tracking task training [59] is also reported though insensitive to amount of assistance given. Mazzoleni et al. [48] on the contrary, proclaimed significant improvement in mean velocity for clockwise CO-PTP movement in both sub-acute and chronic patients with no significant difference between them (i.e. the improvements are similar) although failed to do in previous attempts with smaller samples [46, 47]. In the same nuance, Panarese et al. [62] utilized the mean tangential velocity (MV) of the elbow-shoulder manipulator handle in shape tracing following segmented square (SP) and diamond-shaped (DP) path as shown in Figure 4. Significant increase in MV is reported with curves for different segments were significantly different at the end of the treatment signifying the influence of direction to the course of recovery.

Rohrer et al. [1] reported significant difference between the inpatient (acute) and outpatients (chronic) in mean and peak speed of the end effector. Significant changes are reported in inpatient’s mean speed in comparison to outpatients and moderately correlates to Fugl-Meyer score. An increase of peak speed after PTP movement training [10] is also reported in chronic patient and the authors claimed the increase as expected since the context requires subject to move as fast as possible. Significant correlation of mean and peak speed are reported [35] to FMA-UE, MP, and MSS. The upper range of peak speed and lower range of ratio between mean speed and peak speed overlapped in contribution to predicting the MSS after backward regression analysis. Kahn et al. [14] however opt for a normalized parameter as a supported fraction of speed (FS) is utilized. It is defined as the derivation of distance traveled by the chronic subject’s hand from the starting position, normalized to the same measure for the ipsilateral limb. Improvements in FS for all subjects are reported to be significant across all interventions and across all impairments.

In bimanual task, Sanguineti et al. [59] and Squeri et al. [65] both reported a faster movement as depicted by the increase in average speed at the end of bimanual forward/backward training albeit with no statistical inference. Semrau et al. [19] described through peak speed ratio that many subjects with stroke had difficulty modulating their active arm speed to match the speed of the passive arm.

In summary, mean velocity is able to significantly capture the ease of movement in chronic patients, to distinguish between chronic and sub-acute patients and sensitive to directional changes as well as rotational changes. The peak velocity is able to concurrently validate changes in CMSA. The researchers that utilize both mean and peak speed correlates significantly to FMA, CMSA, MP and MSS indicating concurrent validity with the construct of the clinical score. The use of fraction of speed also able to significantly detect changes however is reported to be insensitive to impairment level.

### Movement smoothness

Post-stroke patients typically present jagged movements appearing as composed of a series of short and rapid sub-movements, representing complete or near-complete stops between each apparent sub-movement [1]. Thus the resulting speed profile has a series of peaks with deep valley in between, consequently produces lower mean speed in comparison to peak speed [48]. Researchers have utilized the movement trajectory, velocity, acceleration and jerk profile as means to capture the smoothness of movement with various parameters signifying the difference. The acceleration metric and jerk metric (which is derived from rate of change of acceleration) for measurement of movement smoothness is obtained from consequences of dynamic behavior of the end-point movement while performing the evaluation task, specifically when frequent start-stop is observed. Patient’s inability to produce accommodative joint torque to maintain muscle tone during interaction results in jagged movement and therefore increases the jerk. While it can be immediately observed through the movement profile, the dynamic measurements are essential to distinguish the direction of generated forces especially when the robot itself prevented such movement [72], thus the information can be “missed”. In recent study, researchers have scrutinized even further to the sub-movement properties to enunciate the variability.

#### The speed metric

With the series of peaks in the speed profile, the significant decrease in number of peaks is recorded in shape tracing activity [13, 62], CO-PTP [1, 18, 48, 50, 51], multi-level PTP [54]. Kahn et al. however report contradicting result later, as insignificant improvement is reported for chronic patients undergoing multi-level PTP [14]. This contradiction suggest that gravitationally influenced task might provide an inconsistent context to evaluate speed peaks in subject’s velocity profile.

The shape tracing activity reveals that the improvement in number of speed peaks in chronic patients is irregardless of segments [62]. The evaluation in CO-PTP activity however unfolds greater insights. Reaching to targets in ipsilateral space has a significantly reduced number of speed peaks than those in contralateral space [50, 51] and left-affected patients have significantly greater number of speed peaks than those of right-affected and healthy controls [18]. The measure also correlates strongly with Bimanual Activity Scale which transfers the improvement in unimanual task to bimanual movements.

Furthermore, the use of speed difference (speed maxima minus speed minima) [18], speed correlation to idealized profile [44] and speed ratio (mean speed divided by the peak speed) [1, 35, 48] also reveals significant improvement to movement smoothness in stroke patients performing CO-PTP activity. Bosecker et al. in earlier study [35] reported a significant correlation of the speed ratio to FMA-UE, MP, and MSS in the study of chronic patients. Contextually, with the increase of velocity; a time shift of the peak speed to the middle of the motion time interval is observed, in compliance with the optimizing minimum jerk-strategy of maximizing smoothness [10]. In chronic subjects, a shift of velocity peak to the center of the time interval is observed, thus tending to approximate a symmetric bell- shaped velocity profile.

While speed difference [18] and correlation measure [44] in chronic patients performing CO-PTP reveals significant difference, Conroy et al. [45] utilizing speed ratio on the contrary, failed to capture statistically significant changes in both CO-PTP and multi-level PTP. Furthermore by performing CO-PTP, only acute [1] and sub-acute patients record significant improvement in speed ratio in comparison to chronic patients [48] albeit weakly correlated to both FMA-UE and Motor Index (MI). While attributing to the difference in rehabilitation robot employed in the study might suggest the effectiveness over the other (KINARM vs. InMotion), the appropriate choice of kinematic parameters might also contribute to the discrepancy in capturing changes in chronic patients.

Rohrer et al. [1] introduces mean arrest period ratio to outline the improvement of both acute and chronic patients in CO-PTP activity as it is common for patients to stop multiple times before reaching the target.Acute patients eventually exhibit significantly greater increases in this parameter albeit weakly correlated to FMA. Vergaro et al. [63] also utilized the movement arrest time ratio in evaluating chronic patients where any movement below 20% of the mean speed is deemed arrest. This indicator is hypothesized to approach zero as training proceeds. Significant decrease is recorded at the end of the treatment.

Overall, most studies reported a significant improvement of speed peaks in chronic population across different activity albeit influenced by target location and lesion area indicating fitness of parameter to reflect changes of movement smoothness. The ratio of mean speed to peak speed shows significant measure in sub-acute patients however ambiguous in chronic subjects. Further studies should consider the evaluation of speed metric in ipsilateral space of planar activity to better gauge the improvement of stroke patients regardless of phases of recovery.

#### The acceleration metric

Mazzoleni et al. [46] reported an increase in mean acceleration albeit not statistically significant in the assessment of planar reaching movement in chronic patients. In another study later, they claimed that higher value in the ratio between the mean acceleration and the peak acceleration illustrate movement smoothness [48]. Statistically significant improvement is reported in this parameter for sub-acute patients; however it is not significant for chronic patients. Unfortunately, the metric shows weak correlation to both Fugl-Meyer scale (UE) and Motor Index (MI) which signifies that the parameter did not reflect the changes that occur in clinical assessment. The findings suggest that the parameter is weak to capture the changes in various phases of stroke recovery and have no concurrent validity to clinical assessment administered.

#### The jerk metric

The smoothness of movement is depicted by trajectory profile that has a smooth bell-shaped curve which therefore minimizes the jerk over the movement time. Researchers have adapted this measure to reflect hypothetically that the recovery of patient post-rehabilitation are closer to those of healthy subjects as jagged movements are less observed.

In CO-PTP activity, Rohrer et al. [1] divide the negative mean jerk magnitude by the peak speed to be utilized as the jerk metric. Outpatients’ significant increase in this parameter indicates increase in movement smoothness. Chang et al. [60] also report a significant within-subject effect. Colombo et al. [13] however reveal ambiguous results for both sub-acute and chronic patients in similar activity. In multi-level PTP, a significant decrease in average norm of jerk across target locations [58] is reported without the influence of target height or location angle while Zollo et al. [10] on the other hand, report a significant decrease in the values of mean jerk magnitude by trajectory length in an unperturbed PTP movement against healthy subjects and significantly correlated to FMA and MP scores respectively.

These studies reveal that normalized measures are more susceptible to significant changes than the mean value itself. Hogan et al. [92] in his review of the use of various jerk parameters in defining movement smoothness suggest the dimensionless measure as it increases monotonically regardless of the overlapping or separation of the sub-movement. Moreover, it reflects changes in movement shape with duration properly than the measurement with units. Furthermore, it captures the multiple speed peaks and period of arrest better than the measure of number of speed peaks or movement duration.

In recent study, Balasubramanian et al. [93] support the use of dimensionless jerk as measure of movement smoothness. They claim that movement smoothness is a measure of signal geometry which is independent of its amplitude and duration, and thus must be dimensionless to be valid. The CO-PTP reaching task assessed on stroke patients with mild and severe hemiparesis as well as healthy person performing force field adaptation reveals that none of the existing measures, whether dimensionless or not, are sensitive to movement smoothness for severe hemiparesis subjects. However their choice of dimensionless jerk, *spectral arc-length metric* is empricially superior as it is sensitive to patients in both phases of stroke recovery, able to avoid the ceiling effect and consistent in comparison to existing jerk measures of movement smoothness.

#### The sub-movement metric

The measure of sub-movement properties are driven from the research done by Woodworth in 1899 [94] that human arm movement is comprised of a sequence of sub-movements. Krebs et al. [95] outline this idea through a repetitive circle drawing in successive increase of speed by a normal subject. The normalized speed profile revealed remarkably consistent pattern and suggested that the movement is characterized by kinematic properties (thus of a number of blended segments), and not temporal.

Rohrer et al. [1] simulate the sub-movement blending by progressively blend two minimum-jerk curves at various states of blending to analyze the sensitivity of the gross movement kinematics. Balasubramanian et al. [96] later use this idea to reflect the temporal coordination of sub-movement using a greater number of sub-movements (up to 5 sub-movements). It is determined by the sum of all maxima detected in normalized frequency spectrum of velocity signal. Smoother movements involve better temporal coordination of sub-movements, thus the lower the maxima the better. The spectral method utilized is able to visualize the trend more intuitively and confirm the suggestion that the decrease in number of sub-movements indicates smoother movement.

In studies on chronic patients, Sanguineti et al. [59] report a significant improvement in number of sub-movements recorded for patients performing outward PTP reaching task with greater improvement for subjects with greater impairment. Similarly, reduction in sub-movement number (of 15 sub-movements) after the PTP movement training [10] are recorded albeit without significance.They reported quite a constant value for sub-movement duration and rate, thus confirming that both of the parameters are intrinsic to patients and independent to pathological level.

In an attempt to discover the relationship of sub-movement to existing clinical assessment, Bosecker et al. [35] decompose the speed profile into support-bounded log-normal sub-movements parametrized by the number of sub-movement, sub-movement duration, overlap, peak and inter-peak interval and shape. They reported significant correlation of all the parameters defining sub-movement to FMA-UE, MP, and MSS and performs better than the gross movement measures albeit not being the strong predictor for the clinical scales. The measures however are not significantly correlated with MAS. This finding signifies that the scrutiny of movement components enhance the concurrent validity of the parameters to clinical scales however should not be used solely for prediction in predictive validation analysis.

While all the studies previously are attempted on chronic patients, the improvement shown in chronic patients with greater impairment might be useful for analyzing sub-acute patients as motor learning and rate of recovery is greater [97]. However, the measure must be supported by other aspects of movement quality for prediction of clinical outcomes.

## Discussion

### The evaluation task

Assessment of quality of movement in stroke rehabilitation helps to enunciate the progress made by patient and especially the contribution made by the intervention to the improvement of subject’s impairment. Considering the fact that the task performed in robot-assisted rehabilitation observed in this review is mainly designed to increase the use of proximal or distal movement or both [2] during intensive training, the outstanding feature is that the training does not involve the use of activity of daily living such as the one addressed in clinical evaluation but rather the artificial task designed to challenge specific joints. While the use of such task is backed by sensorimotor demand and patient’s motivational purpose especially in game-based rehabilitation program [98–100], effort should be made to design the task that mimic the movements involved in the activity of daily living as repetitive practice that can be carried over into daily activities is essential for functional improvement [101]. It is evident from this review that motor improvement is evaluated within the workspace of the task trained, but whether the workspace encapsulate the range of movement in all of daily living activity [102] is still arguable.

Cochrane Review [8] on the effectiveness of electromechanical and robot-assisted arm training concludes that there is evidence of improvement of arm function and strength but not on activities of daily living and that the robotic intervention is highly unlikely to provide better results than therapy provided by human under the same premise of intensity, amount and frequency [3]. Thus, the failure to extend the improvement attained through robot assisted repetitive practice to the daily activities may be attributed not only to the task chosen but also to the set of clinical outcomes that are used to evaluate the functional capacity. Kinematic parameters that have strong correlation in this review are associated largely with FMA-UE which assess the motor function but not activity. Only parameter defining movement planning in bimanual therapy is evaluated with clinical measures specifically assessing activity of daily living (Functional Independence Measure-FIM) and record a significant improvement. Furthermore, since FMA-UE is largely characterized by functional movement (such as active movement of joints/segments in certain range) rather than activity-based movement (such as buttoning shirt etc.), the use Action Research Arm Test (ARAT) which scrutinize the use of upper limb to activity completion as a better replacement has been suggested instead [3]. However, future studies that consider the use of ARAT to correlate the kinematic parameters obtained during assessment should also consider the cultural bias of such tasks (grip, pinch, and grasp) in performing activity of daily living to be valid for population tested.

### The influence of robot characteristics and therapy

It is undeniable that the significance of acquired kinematic parameters may have the influence of the therapy provided by the robots. Parameters representing movement planning are reported from studies using unimanual end-effector robot (ARM-Guide, HapticMASTER, InMotion2, InMotion3), bimanual end-effector robot (BFIAMT), unimanual exoskeleton (T-WREX) and uni/bimanual exoskeleton (KINARM). While T-WREX assists 3D movements in task space, other robots are actually providing planar assistance. The largest clinical study pertaining this aspect is done using KINARM in which both chronic (113 patients) and sub-acute (100 patients) show significant improvement in movement planning and the latter has strong correlation with clinical scores. Other robot that worth mentioning is InMotion2 (84 chronic patients) in which the result shows significant improvement after targeted planar reaching task. The results might suggest that targeted reaching, resistive therapy and bimanual matching helps to improve feed-forward control of stroke patients.

Significant improvements in temporal efficiency is reported from studies using unimanual end-effector robot (BiAS-ADLER), bimanual end-effector robot (MIME,BFIAMT), unimanual exsoskeleton (L-Exos) and uni/bimanual exoskeleton (KINARM). Interestingly, all parameters reported has no strong correlation to any of clinical scales evaluated. This includes the study utilizing KINARM which has the largest patients in comparison to others although significant improvements are observed. This would conclude that the improvement in temporal efficiency using active-assistance which includes both impedance and gravity, as well as passive and resistive therapy may not be transferred to improvements of impairment and functional ability of patients in performing activity of daily living.

Parameters representing accuracy is reported in various targeted task. Studies utilizing ARM-Guide, MIT-MANUS, InMotion2, MEMOS, T-WREX, and BdF all report significant improvements in this aspect. Out of these studies, significant improvement with strong correlation to clinical score is reported from the study on 117 chronic patients using InMotion2 and MIT-MANUS, which both train the subjects on targeted planar reaching in passive, resistive and assist-as-needed mode. All of these robots have passive training in common while InMotion2 is the only robot with assist-as-needed mode in which assistance is given when subject is unable to complete the task by providing force that is time-varied.

Perhaps the most controversial parameter is movement efficacy since its both device and task dependent. Majority of the significant results originates from studies using MEMOS with only one study using KINARM. Interestingly, both robots contrast significantly in the way they are operated. KINARM has impedance control in which it detects the movement of interacting subject and restitutes a force at the point of interaction [103]. MEMOS however is admittance controlled in which robot adjusted its behavior (movement) accordingly to the force input by the user. MEMOS trains patients in active-assisted mode while KINARM does it in active-resisted mode. Nevertheless, subjects under study are able to provide intended result with the parameters chosen.

Parameters that report significant improvement and strong correlation with clinical scores in movement efficiency originate from the studies using InMotion2 as well as InMotion3 which have forearm support. InMotion3 train movements in 3D space, while InMotion train them in planar task. Both robots employ assist-as-needed training in which kinematic parameters are taken as input to control the amount of forces relayed at the tip of the end-effector. Although movement efficiency itself is just a reflection of dynamic interaction of upper limb, the contribution of assist-as-needed training adopted by the robots can be beneficial.

Only one study employs measure of intra-limb coordination in which synergistic movement during circle drawing is studied to reveal the usual kinematic coupling of shoulder and elbow (elbow flexion - shoulder horizontal abduction, elbow extension - shoulder horizontal adduction) observed in chronic patients. However, this study is conducted on 117 patients and report significant improvement as well as strong correlation to clinical scores in parameter selected. It also further reveals that the outcomes of the rehabilitation using InMotion2 on chronic patients support augmentation of existing motor behavior rather than extinction of old abnormal motor synergy [39]. As the training focuses on CO-PTP task that is synergistic in assist-as-needed mode of rehabilitation, further studies should consider the influence of assist-as-needed rehabilitation to motor behavior to confirm the augmentation of the abnormal synergy in chronic patients and its effect to efficiency of the movement. This finding might be helpful to shape the rehabilitation plan suitable for patients in improving their quality of life.

While a lot of measures are presented to evaluate movement smoothness, only studies by L-Exos has both significant improvement and strong correlation to clinical score. It is important to point out that the robot employs gravity balancing and impedance assistance in reaching task which might be useful to decrease otherwise jagged movement observed in stroke patients.

There are no parameters representing range and ease that are able to have significant improvement and establish strong association to clinical parameters. Although it is speculative at this point considering the total number of studies taken into consideration in this review, the current state-of-the-art rehabilitation may not be beneficial to improve the range of movement as required in clinical assessment and parameters representing ease of movement requires additional measure to be a strong predictor to recovery in the upper limb based on this review.

### Kinematic data acquisition

The majority of the kinematic data for the evaluation of patient’s improvement is internally acquired from the robot itself, either through motor encoder [10, 13, 27, 57, 104, 105], tachometer [44], potentiometer [44], electromagnetic sensor [22, 54] or a combination of them [46–48, 96] attached at specific joints under study. While this is the most intuitive solution for robot-assisted rehabilitation system as no external measures are required, care should be taken as the bio-mechanical model of specific robot or electromechanical assistance especially those built with less than seven degrees of freedom are prone to simplifications and assumptions. International Society of Biomechanics (ISB) has defined proper definition of joint coordinate system and rotation sequence for trunk, shoulder, elbow, wrist and hand as natural as possible to normal movement [106]. They further suggest the use of globe method to define shoulder movement rather than clinical rotation sequences such as forward flexion, abduction and rotation which are used by the studies to define their bio-mechanical models in this review. However, there is a promise that the robots may be able to optimally assess the patients if they are able to allow patient to move without perturbations either from internal dynamics [71] or gravitational loading and also maintain the kinematic coupling between the robot and patient [107] throughout the assessment session.

On the other spectrum of assessment, the widely accepted commercial based motion tracker such as VICON [108, 109], Optotrak [110, 111], and Real-Time Motion Analysis [112, 113] are utilized mainly due to their operational accuracy (typically within 0.01 mm). However, the tedious and costly setup of multiple cameras limits the generalization of the system to the robot-assisted rehabilitation. The external measures are imminent for complex evaluation such as in bimanual activities [15]. Furthermore, the overall aspects of the use of compensatory strategies through redistribution of work across the upper limb [37, 114] and the proprioceptive aspects of inter-limb coordination especially in bimanual exoskeleton task require external measures. The overviews of such methods in human motion tracking are published elsewhere [42, 115, 116].

A more cost affective solution such as using webcam and off-the-shelf RGB-D cameras such as Kinect [117] outlines the problem in model fidelity [118], difficulty to assess distal segments [119] including the hand [120, 121], large static error when benchmarked with commercial motion capture [122, 123] and false detection of trunk rotation for compensatory movement [124]. Chen et al. [125] in their survey of depth imagery concluded that the higher resolution body part modelling is required for further research to improve the distal recognition challenges in human action recognition. This supports the findings that model fidelity may need further improvements to influence the quality of the recognition.

Similarly, there is an attempt to use inertial measurement unit (IMU) at the wrist of the unaffected hand presented by [126] to evaluate the bimanual activities using unimanual exoskeleton. Unfortunately, it is incapable of measuring the joint coordination and the proximal movement of the ipsilateral arm thus relying only on end-point measures for quality. It is widely accepted in the stroke community that ipsilateral arm is not fully unaffected [127] and the study on chronic subjects previously reported significant deficits of the ‘unaffected’ arm in regard to gross manual dexterity, fine manual dexterity, motor coordination, global performance and proprioception [128]. Thus, it is substantiated that the measurements on proximal segments of ipsilateral arm for bimanual activity must also be considered to fully understand the extent of stroke impairments.

### Movement quality measures

Parameters defining feed-forward sensorimotor control are pronounced in all PTP and resistive activities as well as bimanual matching for sub-acute and chronic patients and significantly represented by end-point measures. Measures of temporal efficiency should be pre-empted with lesion definition and evaluated in targets located ipsilateral to center of subject’s body to better gauge the improvement. Furthermore, improvement of temporal efficiency is significant for all studies evaluating sub-acute patients, and eminent in PTP activities performed by chronic patients.

Accuracy of end-point measurement significantly reflect improvement in chronic patients but not on sub-acute patients while the efficiency is reported to have been influenced by gravity compensation and type of activity (whether unimanual or bimanual). The studies on efficacy however reflect the difficulties determining parametric contributions of the improvements as only distal measurements are taken into consideration when proximal and distal support are given.

Intra limb coordination parameters are able to capture the synergy in synthetic movement and contralateral to ipsilateral target (left to right for left-affected patients or vice versa) are reported to be more useful to assess abnormal synergy. While loss of proprioception has been identified to produce deficits in intra limb coordination [129], none of the studies apparently does the combination of both in their evaluation.

Parameters defining the abnormality of muscle tone significantly reflect improvement in sub-acute patients, while improvements in chronic patients particularly in range of motion is influenced by nature of the task. Significant results are reported from multi-level PTP and constrained reaching movement, signifying that the gravitationally influenced constrained evaluation task is needed to capture the changes intended. On the other hand, the measure of average velocity as the indicator of ease of movement is able to significantly capture changes in chronic patients, to distinguish between chronic and sub-acute patients and sensitive to changes in rotation and direction.

The speed peak emerge as the significant entity in speed metric defining movement smoothness and influenced by the direction of task completion. The studies reviewed however do not utilize the dimensionless jerk as suggested by Hogan et al. [92] but nevertheless reveal the improvement in movement smoothness in chronic patients, although ambiguously in sub-acute patients.

Overall, kinematic parameters defining movement quality are largely acquired though end-point measurements (either wrist location or robot end-effector) and relies on the specific task that are designed to challenge specific joints or set of joints (shoulder-elbow coordination). Although improvements are presented through various studies, further clarifications of which segment of upper limb that contributes to improvements are needed to better evaluate the course of recovery in stroke patients. It is well known that stroke patients tend to use greater proximal movement to compensate the decrease in functionality of distal segments. However, since these end point measures do not emphasize the segments that contribute to the improvements in parameters evaluated, it is ambiguous whether the improvement is due to the genuine recovery of distal segments or the compensation strategy by the use of proximal segment instead.

Although the functional recovery is the intended outcome of the rehabilitation, the lack of measurement in joint-coordination to the fulfillment of intended task results in the uncertainty of subject’s decision to exploit the joint redundancies to accomplish the task. Only one study in this review presented the intra-limb coordination with significant outcome and concurrent validity to clinical outcome of motor function albeit not to specific measures of activity of daily living. This shows the important of this parameter in defining the movement quality. While the existing study evaluated the intra-limb coordination in circle drawing task, further study should include task that emphasize direction for task fulfillment as directional influence are apparent in other parameters. Furthermore, the concurrent validity to clinical score reported in the number of parameters remains inconclusive due to lack of sub-scores in proximal and distal components in the clinical assessment score. Clinical study on compensatory arm reaching strategies [130] claimed that the increase in shoulder movement in relative to elbow movement was associated with less impairment and greater gains of speed in functional task. Thus it is ascertained that the needs to observe the joint coordination in outlining the synergy to complete the task.

In the same nuance, the exploitation of joint redundancies in task completion also refers to the compensatory strategies employed to attain the goal. With the majority of robot assisted task employ harness to restrain trunk movement which restricts the scapula movement up to 60 degrees to both shoulder flexion and abduction [85], the proximal strategy of task attainment [37] are assumed non-existent and are not evaluated. The interventions that adhere to Brunnsstrom approach [86] are prone to release the harness to allow alternative pattern of motor recovery and use of compensation strategy while those adapting to Bobath [131] strictly prohibited any compensatory movement. However, in both cases the use (or lack) of compensatory strategies must be measured to evaluate the patient’s improvement. Thus, by appropriate measure of inter-joint coordination, the use of either a more distal approach to attain the target or the increase of shoulder movement can be discerned if measurement is available.

Furthermore, robot-assisted therapy offers variation of force inputs either to counterbalance user’s arm during training (active-assisted) or imposing certain force fields to resist the movement in order to increase user’s strength in active-restrained rehabilitation. Thus, analyzing the exchanged force level would be necessary to give further insight on user’s contribution to the quality of movement as the effect of the rehabilitation. Aspects of movement quality such as efficiency and ease can therefore be better understood. The findings from this review may also benefit other research domain such as human motion analysis that studies movement adaptation of healthy person while incorporating force fields.

## Conclusion

In an attempt to assess the quality of patient’s movement in robot-assisted rehabilitation, this review presents the classification of kinematic parameters describing the movement quality according to the weaknesses exhibited by stroke patients. Indeed, the choice of assessment task determines the range of parameters defining movement quality and may provide further insights to the effectiveness of robot-assisted rehabilitation. Beyond the use of external motion capture, the challenge of rehabilitation robot to assess movement quality of stroke patients lies on the ability to counterbalance robot dynamics and gravitational loading as well as maintaining posture alignment during assessment session. If indeed this is difficult to establish in current state-of-the-art rehabilitation robots, the acquisition of movement quality parameters through motion capture without the expensive commercial motion sensing system are still facing several issues such as in establishing appropriate model, unstable distal movement recognition, low processing speed as well as accuracy.

While there is a wide distribution of kinematic parameters to define the movement quality, it is generally used to describe the end-point movement rather than incorporating proximal measurements to characterize the improvement. Furthermore, the parameters representing ease and efficiency for example should not be addressed as purely kinematic parameters as they represent only the consequences of the dynamic interaction between the components of the upper limb. The lack of kinematic measurement of joint synergy in task with directional emphasis is observed, and the measure of compensatory strategies is minimal. Without these measures, the difficulty to differentiate between genuine improvement due to motor recovery or compensated movement is even more apparent. Due to the insufficient correlation studies with standard clinical assessment, the effort to drive kinematic parameters as predictor to the clinical outcomes for better concurrent feedback to the patients is also challenging. Thus, greater effort should be geared towards providing better assessment solution to ensure the validity of continuous assessment from robot-assisted rehabilitation.

## Electronic supplementary material

Additional file 1: Distribution of kinematic assessment describing movement quality in robot-assisted rehabilitation. (PDF 618 KB)
