# Supplementary material for: Assessment of movement quality in robot- assisted upper limb rehabilitation after stroke: a review
Source: J Neuroeng Rehabil. 2014 Sep 12;11:137. doi: 10.1186/1743-0003-11-137 (PMC4180322; doi:10.1186/1743-0003-11-137)
Supplement: Supplementary file 1 — Additional file 1: Distribution of kinematic assessment describing movement quality in robot-assisted rehabilitation. (PDF 618 KB) [file 12984_2014_657_MOESM1_ESM.pdf]

First Author, Year published	Subjects (n)	Phase	Rehabilitation Robot	Evaluation activity	Loss of somatosensation (feed-forward)		Paresis & Loss of somatosensation (feedback)			Loss of fractionated movement		Abnormal muscle tone					
					Plan	Inter-limb coordination	Temporal efficiency	Accuracy	Efficacy	Movement Efficiency	Intra-limb coordination	Range (ROM)	Ease	Smoothness			
														$\dot{x},\dot{\theta}$	$\ddot{x},\ddot{\theta}$	$\dddot{x},\dddot{\theta}$	-sub
**Reinkens-meyer** [128] **2000**	3	Chronic	ARM-Guide	Multi-level PTP	③							③					
**Burgar** [129] **2000**	21	Chronic	MIME	• Shape tracing<br>• Multi-level PTP						③							
**Kahn** [62] **2001**	14	Chronic	ARM-Guide	Multi-level PTP			③	❸				③		❸			
**Rohrer** [1] **2002**	31	Acute (n=12) Chronic (n=19)	MIT-Manus	CO-PTP			① ③						❶	❶❶		❸	
**Lum** [83] **2002**	27	Chronic	MIME	Targeted Reaching								❸*					
**Lum** [63] **2004**	13	Chronic	MIME	Multi-level PTP			❸										
**Daly** [71] **2005**	12	Chronic	InMotion2	CO-PTP				❸						❸			
**Toth** [85] **2005**	8	Sub-acute (n=2) Chronic (n=6)	REHAROB	Therapist guided full ROM slow movement								②③					
**Colombo** [68] **2005**	16	Chronic	MEMOS	Shape tracing				③	❸ ❸❸				❸				
**Kahn** [14] **2006**	19	Chronic	ARM-Guide	Multi-level PTP				❸				❸❸	❸❸	③			
**Lum** [84] **2006**	27	Sub-acute	MIME	Multi-level PTP								❸					
**Colombo** [27] **2006**	19	Chronic	MEMOS	Shape tracing				❸	❸ ❸❸	❸			❸				

$\dot{x},\dot{\theta}$: Speed metric $\ddot{x},\ddot{\theta}$: Acceleration metric $\dddot{x},\dddot{\theta}$: Jerk metric -sub: sub-movement metric

❶ significant improvement in acute patients (P<0.05) ❷ significant improvement in sub-acute patients (P<0.05) ❸ significant improvement in chronic patients (P<0.05)

① insignificant improvement in acute patients (P>0.05) ② insignificant improvement in sub-acute patients (P>0.05) ③ insignificant improvement in chronic patients (P>0.05)

All the indicator has asterisk (*) if the parameter used is reported to have strong correlation to any standard clinical scales.

Multiple symbols of different type in one category indicates different stages evaluated, multiple symbols of the same type in the same category indicates more than 1 parameter used to define the characteristic

First Author, Year published	Subjects (n)	Phase	Rehabilitation Robot	Evaluation activity	Loss of somatosensation (feed-forward)		Paresis & Loss of somatosensation (feedback)			Loss of fractionated movement			Abnormal muscle tone				
					Plan	Inter-limb coordination	Temporal efficiency	Accuracy	Efficacy	Movement Efficiency	Intra-limb coordination	Range (ROM)	Ease	Smoothness			
														$\dot{x},\dot{\theta}$	$\ddot{x},\ddot{\theta}$	$\dddot{x},\dddot{\theta}$	-sub
**Sanchez** [74] **2006**	5	Chronic	T-WREX	• Free reaching<br>• PTP				❸❸	③			③③					
**Dipietro** [39] **2007**	117	Chronic	MIT-Manus InMotion2	Circle drawing				❸*<br>❸*			❸*						
**Chang** [55] **2007**	20	Chronic	BFIAMT	Reach and touch	❸<br>❸		❸									❸	
**Fazekas** [87] **2007**	15	Chronic	REHAROB	Therapist guided full ROM slow movement								③❸					
**Beer** [77] **2008**	5	Chronic	MACARM	Multi-level PTP						③<br>③							
**Colombo** [13] **2008**	22	Sub-acute (n=9)<br>Chronic (n=13)	MEMOS	Shape tracing				②❸	❷❸	❷③			❷❸	❷❸		②<br>③	
**Iwamuro** [54] **2008**	10	Chronic	T-WREX	Multi-level PTP	❸							❸				❸	
**Sanguineti** [56] **2009**	9	Chronic	BdF	Outward PTP reaching													❸
	10	Chronic	BdF	Tracking									❸				
	6	Chronic	BdF	Bimanual forward/ backward reaching		③<br>③							③				
**Squeri** [75] **2009**	4	Chronic	BdF	Bimanual forward/ backward reaching					③				③				
**Nef** [44] **2009**	3	Chronic	ARMin	• Passive mobilization<br>• Active virtual catch & hit								③<br>③③					

$\dot{x},\dot{\theta}$: Speed metric $\ddot{x},\ddot{\theta}$: Acceleration metric $\dddot{x},\dddot{\theta}$: Jerk metric -sub: sub-movement metric

❶ significant improvement in acute patients (P<0.05) ❷ significant improvement in sub-acute patients (P<0.05) ❸ significant improvement in chronic patients (P<0.05)

① insignificant improvement in acute patients (P>0.05) ② insignificant improvement in sub-acute patients (P>0.05) ③ insignificant improvement in chronic patients (P>0.05)

All the indicator has asterisk (*) if the parameter used is reported to have strong correlation to any standard clinical scales.

Multiple symbols of different type in one category indicates different stages evaluated, multiple symbols of the same type in the same category indicates more than 1 parameter used to define the characteristic

First Author, Year published	Subjects (n)	Phase	Rehabilitation Robot	Evaluation activity	Loss of somatosensation (feed-forward)		Paresis & Loss of somatosensation (feedback)			Loss of fractionated movement			Abnormal muscle tone				
					Plan	Inter-limb coordination	Temporal efficiency	Accuracy	Efficacy	Movement Efficiency	Intra-limb coordination	Range (ROM)	Ease	Smoothness			
														$\dot{x},\dot{\theta}$	$\ddot{x},\ddot{\theta}$	$\dddot{x},\dddot{\theta}$	-sub
**Lewis** [29] **2009**	15	Chronic	Haptic MASTER	Voluntary constrained reaching	③		③					❸		③			
				Voluntary free reaching	③		③					③		③			
				Assisted constrained reaching	③		③					❸		③			
				Assisted free reaching	③		③					③		③			
**Vergaro** [69] **2010**	10	Chronic	BdF	Tracking figure-8				❸					❸				
**Dukelow** [58] **2010**	45	Sub-acute	KINARM	Bimanual matching		❷ ❷❷											
**Coderre** [18] **2010**	52	Sub-acute	KINARM	CO-PTP	❷❷ ❷❷ ❷②		❷		❷	❷			②	❷❷			
**Conroy** [51] **2011**	62	Chronic	InMotion2 InMotion (Linear)	• CO-PTP<br>• Multi-level reaching	❸		③	③③					③③	③			
**Mazzoleni** [52] **2011**	17	Chronic	InMotion2	CO-PTP	③			③③					③		③		
**Zollo** [10] **2011**	24	Chronic	InMotion2 InMotion3	Unperturbed & resistive point-to-point	❸*		③			❸*			❸	❸❸		❸	③③ ③
**Squeri** [57] **2011**	1	Chronic	BdF	CO-PTP		③ ③③											
**Abdullah** [70] **2011**	8	Sub-acute	Uni of Guelph Therapeutic Robotic Sys.	Shape tracing				②									
**Johnson** [11] **2011**	7	Chronic	BiAS-ADLER	Drink		③❸	❸						③				
				Pour		③③	❸						③				

$\dot{x},\dot{\theta}$: Speed metric $\ddot{x},\ddot{\theta}$: Acceleration metric $\dddot{x},\dddot{\theta}$: Jerk metric -sub: sub-movement metric

❶ significant improvement in acute patients (P<0.05) ❷ significant improvement in sub-acute patients (P<0.05) ❸ significant improvement in chronic patients (P<0.05)

① insignificant improvement in acute patients (P>0.05) ② insignificant improvement in sub-acute patients (P>0.05) ③ insignificant improvement in chronic patients (P>0.05)

All the indicator has asterisk (*) if the parameter used is reported to have strong correlation to any standard clinical scales.

Multiple symbols of different type in one category indicates different stages evaluated, multiple symbols of the same type in the same category indicates more than 1 parameter used to define the characteristic

First Author, Year published	Subjects (n)	Phase	Rehabilitation Robot	Evaluation activity	Loss of somatosensation (feed-forward)		Paresis & Loss of somatosensation (feedback)			Loss of fractionated movement			Abnormal muscle tone				
					Plan	Inter-limb coordination	Temporal efficiency	Accuracy	Efficacy	Movement Efficiency	Intra-limb coordination	Range (ROM)	Ease	Smoothness			
														$\dot{x},\dot{\theta}$	$\ddot{x},\ddot{\theta}$	$\dddot{x},\dddot{\theta}$	-sub
**Mazzoleni** [59] **2012**	11	Chronic	InMotion2	CO-PTP								③	③				
**Panarese** [67] **2012**	18	Chronic	MEMOS	Square and Diamond path PTP				❸	❸				❸	❸			
**Dukelow** [20] **2012**	100	Sub-acute	KINARM	Bimanual matching		❷ ❷❷											
				CO-PTP	❷ ❷❷		❷							❷			
**Frisoli** [64] **2012**	7	Chronic	L-Exos	Sagittal CO-PTP			❸							❸*			
**Frisoli** [61] **2012**	8	Chronic	L-Exos	Sagittal CO-PTP			❸							❸*			
**Mazzoleni** [53] **2013**	50	Sub-acute (n=25) Chronic (n=25)	InMotion3	CO-PTP	❷ ❸								❷❸	❷❸ ❷③	② ❸		
**Wu** [15] **2013**	53	Chronic	Bi-ManuTrack	Reach and press			③			❸					③		
**Simkins** [89] **2013**	15	Chronic	EXO-UL7	Constrained reaching, Pong-style games								❸					
**Semrau** [19] **2013**	113	Chronic	KINARM	Bimanual matching	❸* ❸*					❸			❸				

$\dot{x},\dot{\theta}$: Speed metric $\ddot{x},\ddot{\theta}$: Acceleration metric $\dddot{x},\dddot{\theta}$: Jerk metric -sub: sub-movement metric

❶ significant improvement in acute patients ($P<0.05$) ❷ significant improvement in sub-acute patients ($P<0.05$) ❸ significant improvement in chronic patients ($P<0.05$)

① insignificant improvement in acute patients ($P>0.05$) ② insignificant improvement in sub-acute patients ($P>0.05$) ③ insignificant improvement in chronic patients ($P>0.05$)

All the indicator has asterisk (*) if the parameter used is reported to have strong correlation to any standard clinical scales.

Multiple symbols of different type in one category indicates different stages evaluated, multiple symbols of the same type in the same category indicates more than 1 parameter used to define the characteristic
